# Osteoarthritic Bony Alterations of Temporomandibular Joint and Relation to Low Bone Mineral Density in Postmenopausal Edentulous Females

**DOI:** 10.3390/dj12080238

**Published:** 2024-07-26

**Authors:** Laura Krumpane, Evija Nikitina, Laura Neimane, Andris Abeltins, Una Soboleva, Anda Slaidina

**Affiliations:** 1 Private Practice “JK Dent”, LV-1048 Riga, Latvia; 2 Institute of Electronics and Computing Science, LV-1006 Riga, Latvia; 3 Department of Prosthodontics, Riga Stradins University, LV-1007 Riga, Latvia; evija.nikitina@rsu.lv (E.N.); una.soboleva@rsu.lv (U.S.); 4 Riga Stradins University Institute of Stomatology, LV-1007 Riga, Latvia; laura.neimane@rsu.lv (L.N.); andris.abeltins@rsu.lv (A.A.); 5 Department of Conservative Dentistry and Oral Health, Riga Stradins University, LV-1007 Riga, Latvia; 6 Department of Orthodontics, Riga Stradins University, LV-1007 Riga, Latvia

**Keywords:** osteoarthritis, osteoporosis, low bone mineral density, cone-beam computed tomography, dual-energy X-ray absorptiometry, temporomandibular joint

## Abstract

This study aimed to evaluate the relationship between osteoarthritic bony alterations in the temporomandibular joint (TMJ) and general bone mineral density (BMD) in postmenopausal edentulous females. Cone beam computed tomography (CBCT) scans for both TMJs were acquired for 80 clinically asymptomatic patients (mean age 72 ± 8.8 y). Both lumbar spine and femoral neck measurements of BMD were performed using dual-energy X-ray absorptiometry (DXA). The most frequently observed bony alterations were flattening of the articular surface (47.5%), sclerosis (41.5%), subcortical cysts (10%), and erosions (5%). Osteophytes were not observed. No statistical differences were observed between those who had or did not have radiological signs of bony alterations according to age or DXA scores. The prevalence of radiological findings of degenerative TMJ bony alterations in clinically asymptomatic postmenopausal females did not confirm a connection with a generally low BMD.

## 1. Introduction

Temporomandibular joint (TMJ) osteoarthritis (OA) is one of the most common diseases of the TMJ [1,2,3]. It is estimated that TMJ-OA is detected in 40% of seniors by microscopic examination and 14% by radiographic evaluation [4]. It is an age-related degenerative disease that occurs more frequently in females than in males [5,6,7] because of its association with hormonal factors, especially estrogen levels, which influence the remodeling of the mandibular condyle [8]. Temporomandibular disorders, including OA, are no longer considered to be solely local disorders but rather are the outcome of multiple risk determinants [9].

OA is characterized by the progressive destruction and loss of TMJ cartilage, followed by the release of degraded proteoglycans and proteolytic enzymes into the synovial fluid. This causes a secondary inflammatory response that results in further degradation of joints [8,10,11]. In the presence of TMJ-OA, there is a decrease in the amount of synovial fluid, which leads to the loss of joint function and joint pain [12]. These changes may be observed in the early stages as condylar flattening on plain radiography or computed tomography. However, in advanced stages radiography may reveal osteophyte formation, subarticular cysts, or condylar sclerosis [3].

Cone-beam computed tomography (CBCT) is the most reliable method for examining TMJ bone tissue components [13,14,15]. Compared to conventional computed tomography (CT) for detecting morphological TMJ changes, it generally results in images of CT-like quality [16]. The advantage of CBCT compared to conventional CT is the lower radiation dose to the patient; it is more affordable and requires less space for its operation. The diagnostic efficacy of detecting osseous changes in the TMJ with CBCT is superior to that of linear tomography, panoramic radiography and MRI [17,18]. CBCT images provide extensive diagnostic and treatment monitoring capabilities, such as disease staging and monitoring of OA progression over time [11,15].

Diagnosing temporomandibular disorders and OA depends on the findings of clinical and radiographic examinations. However, radiological changes in the joints are often observed before symptom onset [19].

Osteoporosis is a skeletal disease characterized by decreased bone mineral density (BMD) and microarchitectural damage, resulting in an increased risk of bone fractures [20,21]. According to international guidelines, all women over the age of 65 (as well as women at risk of developing osteoporosis under the age of 65) should undergo a bone densitometry examination [22]. Osteoporosis and OA are both prevalent conditions associated with aging, with decreased estrogen levels and female sex. Epidemiological data suggests that osteoporosis and OA are inversely correlated; however, there are similarities [23]. When analyzing individual bones, the BMD of the osteoarthritic joints has been reported to decrease, especially in the upper limbs [24]. Several previous papers support the hypothesis that a decreased BMD is associated with increased risk of degenerative changes in the TMJ [25,26]. However, some studies do not find such a relationship [27,28]. The effects of estrogen on the TMJ cartilage and subchondral bone are still under investigation [29], as are the effects of bone mineral density on TMJ-OA. Some articles have compared BMD with temporomandibular disorders—a group of TMJ disorders that also includes OA [30,31,32]; thus, not all subjects have OA. To summarize, the relationship between osteoporosis and OA is complex, so there is need for further research.

This study evaluated the relationship between general bone mineral density (BMD) and osteoarthritic bony alterations in the temporomandibular joint of postmenopausal edentulous females.

## 2. Materials and Methods

### 2.1. Participants

The study included 80 women aged 52 to 86 years (mean age 72 ± 8.8 y) appointed to the Prosthodontics Clinic of Riga Stradins University (RSU) Stomatology Institute between October 2017 and November 2019, because of CBCT examinations due to implant planning. The patients included in the study were postmenopausal edentulous women who had been using conventional complete dentures for both jaws for at least three years according to the same standards made in the same technical laboratory, with an admissible vertical occlusion dimension (VOD). Women with early menopause before the age of 45 years or surgically induced menopause were excluded from the study. None of the participants had diseases that caused secondary osteoporosis (kidney diseases, diabetes, hyperparathyroidism, rheumatoid arthritis, Cushing’s syndrome, thyrotoxicosis, or organ transplantation) or used medications affecting bone metabolism (such as glucocorticoids, bisphosphonates, selective estrogen receptor modulators, calcitonin, and active metabolites of vitamin D). Women who smoked or used alcohol excessively (more than 14 units of alcohol per week) did not participate in the study. None of the individuals had known TMJ disorders, and there were no pain, inflammation, or tumors in the jaw area of the face. The examinations consisted of clinical and radiographic components.

The patients provided informed consent, recorded in the informed consent protocol. The RSU ethics committee approved the research (No. 28/05/10.2017). This study was conducted according to the principles of the Declaration of Helsinki.

### 2.2. TMJ Clinical Examination

Structured interviews were conducted before the radiological examinations. All participants orally answered the Research Diagnostic Criteria for Temporomandibular Disorders (RDC/TMD) and Axis I medical history questionnaire [33]. The survey contained questions regarding the existence and duration of pain in the TMJ region, circumstances in which pain or stiffness occurs, and movements limited by pain. Only clinically asymptomatic patients were included.

### 2.3. Imaging Assessment of TMJ

CBCT (i-CAT Next generation, KaVo Dental GmbH, Germany, Imaging Sciences International, Hatfield, PA, USA) examinations were performed with uniform parameters: 120 kVp, 5 mA, 4 s; voxel size 0.3 mm, field of view (FOV) 230 × 115 mm; dentures in occlusion; and standardized head position. Images were analyzed in the Kavo eXam vision 1.6 (TMJ screen) software (KaVo Dental GmbH, Imaging Sciences International, Hatfield, PA, USA) in the sagittal plane perpendicular and coronal plane parallel to the mediolateral long axis of the condyle. The following radiological findings were used to analyze bone change characteristics in TMJ condylar level: articular surface flattening, subcortical sclerosis, subcortical cysts, surface erosion, and the presence of osteophytes [34].

The images were assessed in a darkened room using a computer with an LCD monitor with a resolution of 1920 × 1200 (single 24.1′ LG monitor FlexScan S2202W; EIZO, Nano Corporation, Hakusan, Japan). Two observers performed a TMJ assessment: a maxillofacial radiologist (L.N.) with more than fifteen years of experience in the field and a general dentist (E.N.) with ten years of CBCT image evaluation knowledge. To determine the compatibility of the analysis, the TMJ evaluation was repeated twice at two-week intervals.

After assessing the criteria, patients were divided into two groups: observing OA and without OA. Each patient’s left and right joints were evaluated. If one or both joints were affected, the occurrence of anatomical bone alterations in patients’ TMJ was registered. In case of similar findings in both TMJs, the most affected joint was analyzed and registered. TMJ flattening and subcortical sclerosis are considered indeterminate signs of degenerative joint disease (DJD), as they could represent aging, normal variation, and remodeling or be precursors to DJD [35]. Subcortical cysts, surface erosion, and osteophytes are signs of osteoarthritic bony alterations. In case of discrepancy between the examiners (five patients with TMJ cases), the images were analyzed repeatedly, and discussions were carried out until an agreement was reached.

### 2.4. Measurement of BMD

To determine BMD, dual-energy X-ray absorptiometry (Lunar DXA DPX-NT, GE Medical Systems, Waukesha, WI, USA) was performed on the lumbar vertebrae (L1-L4) and both femoral necks (mean total hip) at Riga 2nd Hospital. Each patient’s T-score (standard deviations below or above the mean for a healthy 30-year-old adult of the same ethnicity and sex as the subject) was obtained. Experienced specialists performed all examinations.

### 2.5. Statistical Analyses

G* Power version 3.1.9.7 was used to calculate the sample size. Based on the results of a previously conducted pilot study that included 30 women [36], the sample size (80 participants) was calculated, where the study power was assumed to be 80% (type II error), alpha was ≤5% (type I error), and the standardized effect size was 0.8 [37]. A large effect size (0.8) was chosen because changes in the DXA T-scale should be relatively large to change a patient’s diagnosis. These values were considered to be significantly different.

Data were analyzed using IBM SPSS Statistics version 2.0. Descriptive statistics (mean, median, standard deviation, and range) and *t*-tests were used for the statistical analyses. Inter-observer correlation between the examiners and intra-observer correlation between each examiner and the two readings were identified and assessed using the kappa statistic. Values < 0.20 were considered slight, from 0.21 to 0.40 were fair, from 0.41 to 0.60 were moderate, from 0.61 to 0.80 were substantial, and values > 0.80 were excellent [38]. Statistical significance was accepted as *p*-value < 0.05.

## 3. Results

Eighty edentulous females were included in the study. Every subject showed anatomical bone change characteristics in TMJ. A cyst was found in 8 and erosions in 4 CBCT images; thus, 12 patients were considered to have radiological signs of OA. Of the indeterminate signs, the most frequently assessed was flattening, found in 38 (47.5%) patients, and sclerosis in 33 (41.5%) patients. Osteophytes were not detected (Table 1).

**Figure 1:**
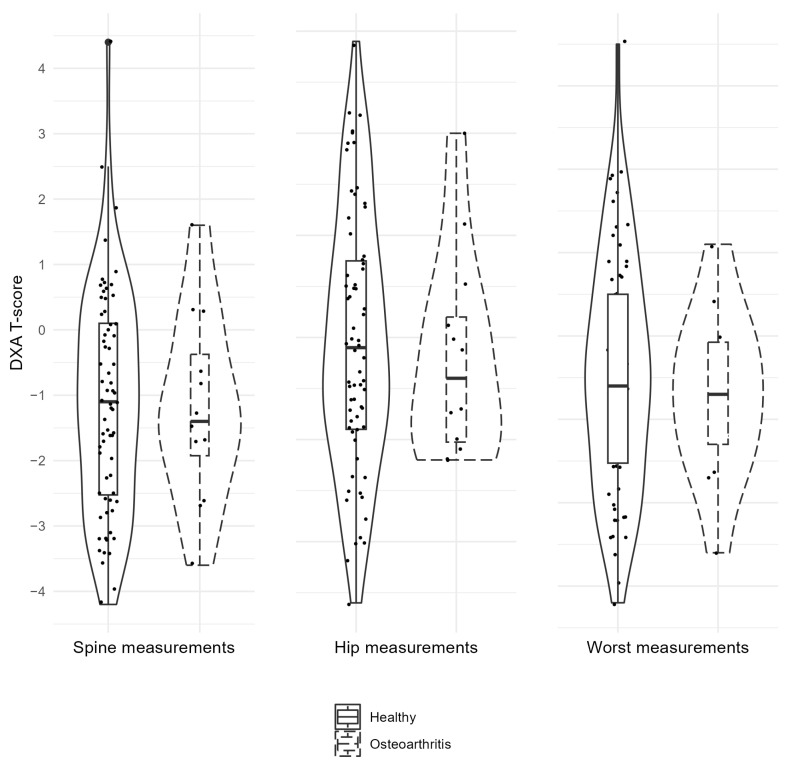
Violin plots for T-scores (DXA) of spine, hips and worst for healthy individuals and individuals with radiological signs of osteoarthritis. DXA—dual-energy X-ray absorptiometry.

There were no statistically significant differences between the groups (observing signs of OA; no signs of OA) in terms of the worst DXA score (*p* = 0.170), DXA spine (*p* = 0.458), or DXA hip (*p* = 0.277) (Figure 1; Table 2) and between groups according to patient age (*p* = 0.856) (Table 2).

Patients were divided into groups based on their radiological findings. No statistically significant difference was observed between those who had or did not have radiological signs of bony alterations according to age or DXA scores (Table 3).

Inter- and intra-observer agreement analyses showed substantial or excellent agreement (kappa index value = 0.796–1.0) regarding the results [38].

## 4. Discussion

In the present study, CBCT was used to determine bony alterations of the TMJ in postmenopausal edentulous females to determine the presence of osteoarthritic changes. DXA was used to evaluate the relationship between BMD and osteoarthritic bone alterations in the TMJ.

The frequency of TMJ-OA in the population (non-patients) is 8.9–36%, but in the patient population is 38–65% [39]. Most often, the joints affected by OA are heavy load-bearing joints, such as the hands, knees, and hips [40,41], but OA can also develop in the TMJ. Internal anatomical alternations are the most frequent reasons for affecting the TMJ, resulting in TMJ disorders, and are most commonly due to disc displacement, followed by inflammatory arthritis and OA [42]. Furthermore, the dimensions of the TMJ spaces may be affected by degenerative condylar changes [43].

In the present study, the most frequent bony alteration was flattening of the articular surface (47.5%), followed by sclerosis (41.5%), subcortical cysts (10%), and erosion (5%); however, no osteophytes were observed. This study was performed on asymptomatic patients; therefore, the results may apply to the general postmenopausal female population. It has been observed that, in the asymptomatic patient group, 15% of controls presented some degree of condylar flattening. Still, this group did not detect surface irregularities, such as osteophytes and erosions [44]. Consistent with the current study, condylar flattening was the most frequently observed bony alteration of the TMJ [19,43,44]. However, condyle articulating surface flattening without evidence of osteophyte formation is not a reliable indicator of OA [45], and subcortical sclerosis of the TMJ can be a sign of age-related TMJ remodeling, or whether condylar flattening or subcortical sclerosis will progress to OA [34,35]. Commonly used radiographic features of joint OA include surface erosion and the formation of subcortical cysts and osteophytes [35,46,47]. This can explain why, when the study was carried out among patients with already diagnosed degenerative joint disease, the most common findings were subchondral cysts (63.3%) and osteophytes (60%) [48], as well as condylar erosion (81.6%) [28].

When panoramic, MRI, and CT examinations for the detection of TMJ-OA were compared, the intra-observer reliability was poor for panoramic images (k = 0.16), better for MRI (k = 0.46), and close to excellent for CT (k = 0.71) [34]. The accuracy of CBCT for TMJ diagnosis has been convincingly demonstrated to be comparable with conventional CT at a lower radiation dose [49]. Owing to the precision of CBCT, this method was chosen for this study. The present study’s inter- and intra-observer agreement analyses showed substantial or excellent agreement of the results, confirming the validity of CBCT.

This study included women who had been postmenopausal for at least three years and were not taking medications affecting bone metabolism. This is because, in postmenopausal women, an increase in bone resorption is detected due to osteoclastic activity, which is generally believed to be related to estrogen deficiency [27]. TMJ bone alterations are more frequently observed in women (73.1%) than in men (55.9%) [48], and therapy-seeking patients with OA are mostly women (85.5% female and 14.5% male) [50]. The higher incidence in women may be explained by the hormonal effects of estrogen and prolactin, which may exacerbate cartilage and articular bone degradation and stimulate a series of immunological responses in the TMJ [51]. Epidemiological studies suggest that osteogenic agents have a positive effect on OA. Postmenopausal women who use hormone replacement therapy may experience fewer structural changes in their joints than those who do not use estrogen [52]. Radiographic clinical trials of strontium ranelate and alendronate in postmenopausal women with osteoporosis showed that this treatment reduced the signs of OA in the spine [52]. However, some authors declared that they did not observe any association between postmenopausal hormone-using women and TMD in their studies [30,31].

The factors contributing to TMJ-OA were limited in the present study, and a homogeneous group of patients was formed. Therefore, only edentulous patients were included in the present study to rule out controversies regarding the position of the TMJ condyle, because trabecular bone morphology, density, and volume of the TMJ condyle are lower in edentulous patients than in patients with teeth [53,54]. Mechanical factors can also cause changes in TMJ structure [8,55,56]. The most common mechanical stress-related factors include functional overload, parafunction, trauma, unstable occlusion, or increased joint friction [8,11]. Loss of the occlusal relationship may lead to degenerative changes in TMJ morphology and bone mass density [54]. Bruxism and unilateral chewing cause joint microtrauma, provoking OA [56].

When processing the present study’s data, no statistically significant differences were observed between BMD, age, and TMJ-OA. Osteoporosis and OA are both prevalent conditions associated with ageing [27]. In our study there was no statistical difference in age between OA and NonOA groups. Reasoning for these results could be the small group of patients with signs of osteoarthritic changes in TMJ. Additionally, our study group consisted of elderly patients (mean age 72 ± 8.8 y), thus there was limited age range. Similar to current research, no relationship has been found between BMD and TMJ OA [27] and radiological degenerative joint findings related to TMJ-OA [28]. However, when patient groups with and without DJD were compared, there was a higher prevalence of self-reported osteoporosis or osteopenia (50.5%) in patients with the disease than in patients with normal TMJ (40%) [48], but this is not a reliable statement for conclusions about BMD correlation with OA, because osteoporosis was self-reported and not clinically examined. Furthermore, compared with a control group without OA, older postmenopausal women with radiographic knee and hip OA had significantly lower spine and hip BMD [57]. There is a higher occurrence of osteoporosis in the lumbar spine in men and women with TMD than in the control group [32]. In addition, it has been reported that radiographic changes in the TMJ (erosion, flattening, and osteophytes) correlate with low levels of a bone formation marker, fragments of Type I collagen telopeptide, that are significant for normal BMD [26], but this should be perceived with caution because only one marker does not confirm the diagnosis of osteoporosis.

In the current study, a tendency was observed between erosion of the TMJ and femoral neck DXA (*p* = 0.095). However, this should be interpreted with caution because only four of all of the patients presented with erosion, which was observed only in the femoral neck.

In order to form as homogeneous a group of patients as possible, strict inclusion criteria for this study were developed. Unfortunately, this means that the results must be interpreted with caution, because they are more difficult to apply to the general population. The main limitation of this study is that it was performed on asymptomatic patients; therefore, it differs from other studies that included patients diagnosed with OA. Another limitation is the small group of patients who had osteoarthritic bony alterations in TMJ. To support our conclusions, additional studies should be conducted on male patients, dentulous patients, subjects of different age groups and divergent conditions of onset of menopause. Further research should be conducted to determine whether a connection exists between estrogen deficiency and TMJ erosion in postmenopausal females.

## 5. Conclusions

The most frequently observed TMJ bony alteration in clinically asymptomatic postmenopausal females is condylar flattening of the articular surface. The prevalence of radiological findings of degenerative TMJ bony alterations did not confirm the association between age and low BMD in clinically asymptomatic postmenopausal females.

## Figures and Tables

**Table 1:** Number of radiographic bony alterations in TMJ.

	Overall N (%)	OA N (%)	NonOA N (%)
Indeterminant signs of OA			
Flattening	38 (47.5%)	6 (7.5%)	32 (40%)
Subcortical sclerosis	33 (41.3%)	6 (7.5%)	17 (21.3%)
Signs of OA			
Subcortical cyst	8 (10%)	8 (10%)	0
Erosion	4 (5%)	4 (5%)	0
Osteophytes	0	0	0

N—number of findings, OA—radiological signs of osteoarthritis, NonOA—no radiological signs of osteoarthritis.

**Table 2:** Characteristics between groups observing and not observing signs of OA and age, DXA results.

Factor (N)	Descriptives	DXA Spine	DXA Hip	DXA Worst	Age
OA (12)	Mean (SD)	−1.2 (1.5)	−1.2 (1.0)	−1.7 (1.0)	72.3 (10.1)
NonOA (68)	Mean (SD)	−1.1 (1.7)	−1.0 (1.2)	−1.5 (1.4)	72.0 (8.7)
	CI, 95%	−1.0; 1.1	−0.6; 0.9	−0.7; 1.0	−5.9; 5.2
	*p*-Value	0.458	0.277	0.170	0.856

N—number of findings, OA—radiological signs of osteoarthritis, NonOA—no radiological signs of osteoarthritis, DXA—dual-energy X-ray absorptiometry, CI—confidence interval.

**Table 3:** Characteristics between radiological findings of bony alterations and age, DXA scores.

Factor	Positive/Negative Finding (N)	Descriptives	DXA Spine	DXA Hip	DXA Worst	Age
Flattening	Positive (38)	Mean (SD)	−1.1 (1.5)	−1.0 (1.3)	−1.5 (1.2)	73.1 (8.1)
	Negative (42)	Mean (SD)	−1.1 (1.7)	−1.1 (1.1)	−1.6 (1.3)	71.1 (9.5)
		CI, 95%	−0.8; 0.7	−0.7; 0.4	−0.7; 0.5	−6.0; 1.9
		*p*-Value	0.721	0.302	0.819	0.447
Subcortical sclerosis	Positive (33)	Mean (SD)	−1.4 (1.5)	−1.1 (1.1)	−1.7 (1.4)	70.8 (9.3)
	Negative (47)	Mean (SD)	−0.9 (1.7)	−1.1 (1.3)	−1.5 (1.3)	72.9 (8.5)
		CI, 95%	−0.2; 1.3	−0.5; 0.5	−0.4; 0.8	−1.8; 6.2
		*p*-Value	0.685	0.347	0.937	0.555
Subcortical cyst	Positive (8)	Mean (SD)	−1.4 (1.0)	−0.8 (1.1)	−1.5 (0.9)	71.8 (11.2)
	Negative (72)	Mean (SD)	−1.1 (1.7)	−1.1 (1.2)	−1.6 (1.4)	72.1 (8.6)
		CI, 95%	−0.9; 1.5	−1.1; 0.7	−1.1; 0.9	−6.3; 6.9
		*p*-Value	0.104	0.428	0.199	0.582
Erosion	Positive (4)	Mean (SD)	−0.8 (2.3)	−1.8 (0.6)	−2.2 (1.1)	73.5 (8.9)
	Negative (76)	Mean (SD)	−1.1 (1.6)	−1.0 (1.2)	−1.5 (1.3)	72.0 (8.9)
		CI, 95%	−2.0; 1.3	−0.4; 2.0	−0.6; 2.1	−10.6; 7.5
		*p*-Value	0.364	0.095	0.342	0.681

N—number of findings, SD—standard deviation, DXA—dual-energy X-ray absorptiometry, CI—confidence interval.

## Data Availability

Data supporting the conclusions of this study are available upon request from the corresponding author (L. Krumpane).
